# Perinatal Palliative Care: Cultural, Spiritual, and Religious Considerations for Parents—What Clinicians Need to Know

**DOI:** 10.3389/fped.2021.597519

**Published:** 2021-03-30

**Authors:** Victoria J. Kain

**Affiliations:** Griffith Health, Griffith University, Southport, QLD, Australia

**Keywords:** perinatal palliative care, parents, religious needs, cultural needs, spiritual needs

## Abstract

For perinatal palliative care (PPC) to be truly holistic, it is imperative that clinicians are conversant in the cultural, spiritual and religious needs of parents. That cultural, spiritual and religious needs for parents should be sensitively attended to are widely touted in the PPC literature and extant protocols, however there is little guidance available to the clinician as to how to meet these needs. The objective of this review article is to report what is known about the cultural, spiritual and religious practices of parents and how this might impact neonates who are born with a life-limiting fetal diagnosis (LLFD). The following religions will be considered—Islam, Buddhism, Hinduism, Judaism, and Christianity—in terms of what may be helpful for clinicians to consider regarding rituals and doctrine related to PPC. Data Sources include PubMed, Ovid, PsycInfo, CINAHL, and Medline from Jan 2000–June 2020 using the terms “perinatal palliative care,” “perinatal hospice,” “cultur^*^,” and “religiou^*^.” Inclusion criteria includes all empirical and research studies published in English that focus on the cultural and religious needs of parents who opted to continue a pregnancy in which the fetus had a life-limiting condition or had received perinatal palliative care. Gray literature from religious leaders about the Great Religions were also considered. Results from these sources contributing to the knowledge base of cultural, spiritual and religious dimensions of perinatal palliative care are considered in this paper.

## Introduction

Perinatal palliative care (PPC) is a specialized branch of pediatric palliative care that considers an interdisciplinary strategy for the care of newborns with life-limiting or life-threatening conditions. The World Health Organization defines palliative care as the “primary goal for the provision of a good quality of life for those with life-threatening diseases.” This expands to palliative care for children and infants as a specialized field that extends to the family of children and infants. The WHO has set up principles of palliative care for children, and it includes the following points: “(a) Complete care of the infant must be taken including mind, soul, and body; (b) Moral support to the family should be provided; (c) Palliative care should start when the decision for not providing any more intensive care has been made; and (d) Care should be implemented even when resources are limited” (1).

For PPC to be truly holistic, it is imperative that clinicians must be conversant in the cultural, spiritual, and religious needs of parents. The need for cultural, spiritual, and religious considerations to be sensitively attended to is widely touted in the literature on PPC and extant protocols; however, there is little guidance available to the clinician on meeting these needs.

In recent times, most life-limiting fetal diagnoses (LLFD) are made during the prenatal period at a time that should represent hope and joy for parents and families. When faced with an LLFD, however, these hopes and dreams of a healthy child fade. It is at the margins of life and death that people consider the existential questions of life and their spirituality, and, at times, they may even question their religious beliefs. The death of their child will lead to maladaptive grief, long-term diminished quality of life, and symptoms linked to psychological morbidity if parents are unable to reconcile these very personal needs (2). Religious, cultural, and spiritual beliefs can bring profound comfort and healing for parents faced with an LLFD, and the majority of parents want the healthcare team to have the sensitivity and skills to discuss and tend to these needs (2).

While it is not possible for the clinician to be knowledgeable about all religious tenets and cultural norms encountered in practice, having the knowledge of the most common religions and how these religious tenets apply to infants with an LLFD or that are born at the margins of viability can assist parents greatly. Sadeghi et al. (3) identified that belief in the force of the supernatural, the need for comfort of the soul, and human dignity for the newborn were the important dimensions for parents of the infants who were not expected to survive (3).

The large variations in religious practice are known from the outset, and it is beyond the reach of this review to identify and accept all of these variations exhaustively. The objective of this review is to report on what is known about the cultural, spiritual, and religious practices of parents and how this might impact the neonates who are born with an LLFD. The following religions will be considered in terms of what may be helpful for clinicians to consider regarding rituals and doctrine related to PPC: Islam, Buddhism, Hinduism, Judaism, and Christianity.In addition, cultural and spiritual needs will be addressed.

## Materials and Methods

Data sources for this review included PubMed, Ovid, PsycInfo, CINAHL, and Medline. The data from January 2000 to August 2020 were collected using the terms “perinatal palliative care,” “perinatal hospice,” “cultur^*^,” “spiritual^*^,” and “religiou^*^.” The inclusion criteria included all empirical and research studies published in English that focused on the cultural, spiritual, and religious needs of parents who opted to continue with their pregnancy, where the fetus had a life-limiting condition or had received PPC. Results from these studies contributing to the knowledge base of cultural and religious dimensions of PPC are considered in this review. Table 1 provides details of the types of papers included and their level of evidence.

**Table 1:** A summary of reviewed studies.

**Authors/year**	**Purpose**	**Method (study design, sample, and data collection)**	**Main findings**	**Level of evidence^1^**
Ayed and Ayed (4)	To provide an overview of palliative care and challenges faced by the health care system during treatment of Muslim infants.	Debate paper based in a neonatal unit in Kuwait City.	The questions posed by medical advances related to fetal and newborn palliative care require a degree of interpretation and application of the Quran by authoritative teachers (Imams).	C
Boss et al. (5)	What categorizes parental decision-making regarding delivery room resuscitation in terms of “religion,” “spirituality,” and “hope.”	Qualitative multicenter study; Parents of infants who had died as result of prematurity or congenital anomalies. Interviews and maternal medical charts.	Some parents felt that they had not made any decisions regarding resuscitation and instead “left things in God's hands.” These parents typically were documented by staff members to “want everything done.” Parents and physicians had different interpretations of what was discussed and what decisions were made.	B
Caulfield et al. (6)	To evaluate trends, outcomes and characteristics of newborn infants of Catholic parents baptized over a 15-year period in an Irish maternity hospital.	Retrospective study. Patients were identified from the “register of baptisms” from the years 2002–2016.	A gradual decline of emergency baptisms over the 15-year period was reported. Maternity hospitals and neonatal units need to have access to emergency baptism service or other equivalent “spiritual blessings” as appropriate to the faiths followed by the family in an emerging multi-faith population.	B
Chichester and Wool (7)	Explores feeding issues for infants who are not expected to survive across a variety of cultures and religions: Amish, Judaism, Hindu, Muslim, African American, Hispanic-Latino and Chinese American.	Discussion paper.	Customs, beliefs and religious laws and ceremonies often involve food and are deeply connected with events, such as birth and death.	C
Das (8)	Belief in reincarnation is widespread amongst Hindus. A newborn is not a new creation, or a by-product of its parents' chemistry—it is an eternal soul continuing its progression of lives within the material world in a new body.	Best-practice guidelines.	Palliative care welcomed for newborns not expected to survive, however Hindus are wary of any intervention that might hasten death or cause it artificially. The Hindu ideal is to remove all active treatments and allow death to come to the baby in its own time.	C
El Sayed et al. (9)	To explore the challenges for trainees when EOL decisions are undertaken and to encourage them to reflect on how they might influence decision-making.	In-depth, semi-structured interview guide developed to address trainees' physician beliefs, attitudes, preferences and expectations regarding discussions of EOL neonatal care.	Dealing with different cultures and an inability to interact effectively with people of different cultures was identified as a barrier.	B
Hawthorne et al. (10)	To examine differences in parents' use of spiritual and religious coping practices by gender, race/ethnicity, and religion at 1 and 3 months after infant/ICU death.	The Spiritual Coping Strategies Scale was used to measure religious and spiritual coping practices, separately. 165 bereaved parents, 78% minority.	Black non-Hispanic mothers used more religious coping practices at 3 months than White non-Hispanic mothers. Protestant and Catholic parents used more religious coping practices than the “no” and “other” religion groups at 1 and 3 months. Within the 30 mother–father dyads, mothers reported significantly greater use of religious coping practices at 1 and 3 months and spiritual coping practices at 3 months than fathers.	B
Kain (11)	To examine the notion of being born dying and karma for Buddhists and non-Buddhists (given that karma is a belief held by both).	Discussion paper.	Spirituality and culture go hand in hand for Buddhists, and Western medicine is beginning to acknowledge the importance of spirituality in healthcare. Spiritual care for dying infants and their families is an essential component of this type of care and is not only the domain of chaplaincy services but of the entire interdisciplinary team.	C
McGuirl and Campbell (12)	To provide a foundation about prominent religions so that clinicians can better understand and assess the potential religious/ moral complexities that families face in the process of making treatment decisions for their infants, including decisions regarding withholding and/or withdrawing active intervention.	Discussion paper.	Knowledge of prominent religious beliefs can be helpful as clinicians enter end of life conversations with parents. Clinicians can use this information to better understand a family's perspectives and strongly held views and can foster clerical and other spiritual support to assist families in exploring, detailing and clarifying their beliefs, values and goals.	C
Raingruber and Milstein (13)	To identify which interactions with health care providers were and were not helpful. Parents discussed what it was like to have an infant with a life-threatening illness and what helped them to cope.	Interpretive phenomenological investigation; purposive sample of 7 parents.	Parents indicated that they benefited from identifying “circles of meaning,” or ways in which their infant touched another life. They appreciated hearing from health care providers that they felt close to their infant.	B
Rosenbaum et al. (2)	To increase practitioners' awareness of spiritual and existential distress and to provide strategies to address such needs, particularly at the end of life.	Literature review of the spiritual care literature and narratives of parents who have experienced the loss of their baby in our NICU.	It is important to support parents through loss by providing comprehensive care that focuses not only on the infant's physical needs, but also addresses parents' and families' spiritual, religious, and existential needs.	C
Sadeghi et al. (3)	To explore the spiritual needs of families in Iran at the end of life and through bereavement in the NICU.	An exploratory qualitative study using purposeful sampling and semi-structured interviews with 24 participants.	Data analysis revealed three main themes: spiritual belief in a supernatural power, the need for comfort of the soul, and human dignity for the newborn.	B
Seth et al. (14)	To describe the role structured religion and spirituality plays in Latinas' daily lives and to evaluate how religiosity and spirituality influences health care decisions, specifically in prenatal diagnosis.	Cross-sectional qualitative study. Semi-structured interviews with 11 women to describe religious beliefs and thoughts while considering the option of amniocentesis for prenatal diagnosis.	Latino women found their religious and spiritual beliefs comforting, providing validation for their decision regarding prenatal diagnosis, as opposed to a direct influential factor in their decision-making process. Their spiritual faith helped maintain optimism even when faced with the complexity of the amniocentesis decision-making process.	B
Shukla et al. (15)	To identify and formulate recommendations regarding challenges faced while considering redirection of care for Muslim infants based on experiences of neonatologists.	Cross-sectional survey using a web-based questionnaire: Neonatologists practicing in countries with predominantly Muslim population (Kuwait, Oman, Saudi-Arabia and Egypt).	Redirection of care consideration for Muslim infants has many socio-cultural and religious barriers. Comprehensive ethical codes conforming to Islamic and legal standards are required to aid decision-making.	B

1 Level of evidence

^A^Body of evidence can be trusted to guide practice

^B^Body of evidence can be trusted to guide practice in most situations

^C^Body of evidence provides some support for recommendation(s), but care should be taken in its application

*^D^Body of evidence is weak, and recommendation must be applied with caution*.

In addition to these published papers, sources discussing the principles of the Great Religions were also included to provide a background about religious doctrine, beliefs, and current global demographics.

## Discussion

### Religious Considerations for Parents

We live in a multicultural, multi-faith world, where religion is seemingly more important than it has ever been, with four out of five people identifying as belonging to an organized faith (16). Christianity is the fastest growing religion, with Islam and Hinduism also experiencing global growth (16). Medicine, in particular, does not accept religious concepts and practices as part of the medical model (17); however, it is increasingly important in considering how faith intersects with the biomedical and psychosocial model of health. Having one's religious and spiritual beliefs recognized brings comfort and may also promote optimism, even when facing a complex prenatal diagnosis with uncertain outcomes (14).

Religion is somewhat simpler to define than spirituality. It considers beliefs, practices, and rituals and may also include beliefs about spirits, both good (angels) and bad (demons). Religion can be observed either publicly, as part of a community, or privately, and often as both (18). It is estimated that 78% of Americans have belief in God, with another 15% believing in a “higher power” or something “bigger than themselves.” In terms of prayer, 84% of Americans believe that their chance of recovery is increased by the act of praying for the sick; 79% believe in the presence of “miracles”; and 72% perceive that God can cure incurable conditions (2). Having religious beliefs can assist people by bringing some order to life when events make life untenable with the offering of rituals, traditions, and guidance. Further, the concept of an “afterlife,” or existential heaven, may bring great comfort to parents faced with an infant with an LLFD.

In a discussion paper, McGuirl and Campbell (12) claimed that knowledge of the most common religious beliefs is helpful for clinicians in having end-of-life conversations with parents who are making decisions for their children, including the decision on withholding and/or withdrawing from active involvement in interventions. However, there are also studies that associate religious beliefs with an increased propensity toward life-prolonging care that may prolong the suffering of infants with life-limiting conditions (2).

In the Southeast and East Asian community, religious and spiritual values may also directly affect the pregnancy decisions linked to genetics. Leung et al. (19) stated that while Chinese women in Hong Kong generally support the termination of pregnancy in the early stages for chromosomal defects and non-medical reasons, religious context is a major contributor to the negative attitudes toward termination. Notably, most women in the study also accepted that for an undesired fetal gender, termination should not be performed. Meanwhile, the health values of some strongly traditional Southeast Asian groups concentrate on the unity of mind and body and may clash with the biomedical model practiced in the United States. Surgery, for instance, is considered a breach of the “conscience”; blood is considered irreplaceable once drawn; and prenatal treatment is considered unnecessary because pregnancy is not considered a disease (20).

It is critical that clinicians are aware of the importance of inquiring about the social and religious affiliations of a patient, as these do not always correlate with the specified religious affiliation of the patient and these are important guiding principles in perinatal decision-making.

#### Islam

Islam impacts all aspects of daily living: from eating to clothing to health practices. Islam has been pursued for more than 1,400 years, originating from Mohammad in Mecca, Arabia, who is believed to be the last prophet sent by God (*Allah*, translated literally as “God”). Clinicians should be aware of the Five Pillars of Islam and work with families to understand how this belief may influence their decision-making when having a baby with an LLFD. For Muslims, the Five Pillars are five broad principles that allow them to live a good and responsible life. These include the following: The Declaration of Faith (*Shahada*); praying five times a day (*Salat*); giving charity (*Zakah*); fasting during the month of Ramadan (*Sawm*); and a pilgrimage to Mecca at least once (*Hajj*). It is important that healthcare providers are cognizant and do not confuse Islamic traditions with cultural traditions.

Comprised of 24.1% of the global population (21), Muslims believe that Allah is in control of both the beginning and the end of life. All outcomes, including death, are predetermined by Allah (21). The teachings of their holy book (the Quran) influences healthcare practices and the pivotal moments of life, such as birth, death, and illness (4). Table 2 provides guidance to clinicians caring for infants of Muslim families.

**Table 2:** Islam—what clinicians need to know.

**For the infant**	**For the parents and family**
• Breastfeeding is encouraged: a Muslim child has the right to be breastfed and raised “with kindness and respect.” • Anything that hastens the death of the baby, including withholding sustenance is unacceptable because the time of death is predestined by Allah. • Suffering can be interpreted as a test of faith; however, pain relief is considered acceptable. • The dying infant needs to face east, and to die facing east. • Muslims do not like to die in hospitals—the same may be true for infants and this requires discussion with parents. • Muslim adults with terminal illness prefer to stay at home with their families until they die—again, discussion with parents is required for infants. • Muslims may not consider morphine a treatment option and may believe more in Allah's potential to heal and reduce pain then on conservative medicines (i.e., Allah has the power to cure ailments.) • The burial (if the baby is viable) should normally take place as soon as possible. • Upon death, enshroud the baby's body in a white cotton or linen cloth.	• Prayer occurs five times a day: dawn to sunrise, noon, afternoon, sunset, and evening. • Prayers are performed facing east. • Muslim law permits termination of pregnancy only if continuing the pregnancy seriously impacts the mother's life. • After 19 weeks of gestation, Muslim law accepts maternal health as the only reason for terminating the pregnancy. • Muslim parents may lean more toward following the Quran rather than seeking medical advice. • Parents may be less likely to question health care providers about their child because this is interpreted as a sign of mistrust. • Muslim families may not like to be reminded of their child's illness repeatedly, or that their child may die due to a LLFD. • The Quran provides reassurance that the baby will be received into paradise and is increasingly consulted as death approaches.

*Ayed and Ayed (4)*.

For practitioners of Islam (Muslims), the pervasive belief is that curative medical interventions come from Allah, and healthcare professionals are the medium for delivering the will of Allah. The Quran provides guidance and is considered the source of healing for psychological and spiritual distress. If family members, including infants and children, require surgical or medical measures, the Quran can be used as an addendum to resolve theological, social, and cultural needs (4).

To facilitate decision-making, rigorous ethical codes are required that adhere to the Islamic social and legal standards (15). When an LLFD is made, the Quran states quite clearly that a pregnancy should not be terminated for reasons that might include financial fears or fears that the parent/s will not be able to care for their infant after birth. However, when considering extremely premature infants born at <25 weeks of gestation (12), the “legal opinion” of the Islamic society is that two specialist physicians need to be involved in the decision-making, and while for some Muslims the sole decision-maker might be the physician who is treating the infant, the parents should always be involved in the decision-making with as much autonomy afforded to them in their decision-making role. The treating physician should, however, freely consult with other pediatricians/neonatologists and caregivers.

The steps of the legal process following the diagnosis of an LLFD according to the Islamic society are as follows: confirmation of lethal malformations by ultrasound and/or chromosomal analysis; approval of the malformation by at least two neonatology and perinatology specialists; documentation of the type of malformation in the medical records of the mother; obtaining the written consent from parents or their delegates; termination of the pregnancy is permitted if the gestational age is longer than 19 weeks, but only if the continuation of the pregnancy is expected to result in the death of the mother and if fetal death *in utero* is confirmed. The infant can receive PPC if born alive (4).

In summary, clinicians need to take care in exploring with families the role of their religion in decision-making and to have some awareness of the teachings and beliefs that guide their daily lives. An understanding of their religious needs provides a framework for discussion and allows the healthcare team to support the parents through difficult decision-making processes (12).

#### Buddhism

Buddhism is a faith that was established more than 2,500 years ago in India by Siddhartha Gautama (“the Buddha”). Comprised of 6.9% of the global population, Buddhists recognize suffering as central to human existence. Suffering is unavoidable and life-limiting illness serves to prompt reflection on the ultimate meaning of life. Death is associated with rebirth (reincarnation), and having serene surroundings at the time of death and during the dying process is important to the dignity of the person and for their rebirth (21). Although Buddhists believe that death is a part of life, the religion is not somber, but it is full of serenity, hope, and wisdom, but recognizing that suffering is unavoidable, just as death is unavoidable. Central to this is a belief in *karma* (as is also in Hinduism), or the sum of one's actions (and inactions) in their current and previous states of existence. The sum of these karmic actions decides their fate in future existences, influencing how they will be reincarnated and what they will be reincarnated as. Karma, however, is not isolated to Hinduism and Buddhism: in America, 27% of the population were reported to have belief in reincarnation and “some form of karma” (11). In 2014, Kain explored the notion of being born after dying and karma for Buddhists and non-Buddhists, in a discussion paper, reported the dilemma of the karmic “baggage” of being born after dying. Because Buddhist scripture does not delineate at which point death occurs along the continuum, whether at the beginning of life or at the end, Buddhist parents can take comfort by realizing that their infant being reborn as a human suggests that their child already had a degree of untarnished karmic “baggage.” Buddhist traditions accept human birth as a beneficial rebirth since the conditions for a sentient being to attain enlightenment are ideal only as a human being, which is the ultimate objective for the Buddhist practitioner (11).

Table 3 summarizes what clinicians need to know in terms of caring for infants of Buddhist families, especially for both the infant and the parents.

**Table 3:** Buddhism—what clinicians need to know.

**For the infant**	**For the parents and family**
• As in Hinduism, the illness of the baby is the result of past and current actions (karma). • At death, Buddhist's believe that the person will go through a process known as “samsara,” or reincarnation. Ensuring a serene setting at the time of death will be important. • Buddhists may not practice specific death rituals, however, rituals that do take place focus on helping the baby achieve a better station in their next life. • In Buddhism, burial and cremation are both practiced, so it is important not to make assumptions of this. • When creating an environment of serenity, white or yellow flowers are acceptable; red flowers are to be avoided as they symbolize happiness. • There are no rules or specific timeframe that determines when the burial or cremation of the baby should take place.	• Parent's will believe their baby could be reborn (reincarnated) as a god, demigod, human, animal, “hungry ghost” or “hell creature,” depending on the baby's karma and actions during their brief life. • The parents may wish to place images of Buddha and flowers near their infant to maintain a sense of calm in the face of death. • A calm, serene environment helps to maintain the focus on religious thoughts and the “good deeds” performed during the infant's short life. • Parents may also consider the karma of their infant, and their own karma when faced with the death of their infant.

*Kain (11) and Lewis and Foley (21)*.

Essentially, when the death of their infant becomes imminent, Buddhist parents need to be able to focus on caring for the spiritual state of the infant rather than unnaturally prolonging the life of their child, as this will encourage a good rebirth. It is important for medication to not interfere with consciousness. When caring for Buddhist families, the clinician will benefit from demonstrating the “right understanding” (this is an appreciation of the four noble truths: the truth of suffering; the truth of the cause of suffering; the truth of the end of suffering; and the truth of the course that frees us from suffering) and an understanding of mindfulness (the whole-body-and-mind awareness of the present moment), which in turn creates good karma for the child and encourages the well-being of the child. In both its present state and as a living being, this will benefit the child in the current life and for the next life to come (11).

#### Christianity

Christianity is a monotheistic Abrahamic faith. Its ideology is based on the life and teachings of Jesus Christ of Nazareth, practiced by over 30% of the global population. Its followers, known as Christians, include many denominations: in the Western world, Roman Catholic; Protestant (Adventist, Anabaptist, Anglican, Baptist, Calvinist, Evangelical, Holiness, Lutheran, Methodist, and Pentecostal); in the East, Eastern Catholic; Eastern Orthodox; Oriental Orthodox Church of the East (Nestorian); and in Nontrinitarian, Jehovah's Witness; Latter Day Saint; and Oneness Pentecostal.

In Christianity, Jesus is considered the savior, and although beliefs vary between sub-denominations, most Christians view illness as a natural process of the body and even as a testing of their faith. While the death of an infant represents profound loss, most Christians view death as being part of the will of God (7). Table 4 provides guidance for clinicians caring for infants of Christian families, especially for both the infant and the parents.

**Table 4:** Christianity—what clinicians need to know.

**For the infant**	**For the parents and family**
• Baptism by water is considered necessary for salvation using a formulaic prayer, depending upon the sub-denomination. • For infants who die before baptism, the Order of Christian Funerals contains a special rite for these infants.	• Christians of all sub-denominations often have a belief in miracles, especially through prayer. • There is no firm, detailed Dogma regarding consolation of parents, however, parents will find comfort in the simple faith that God wants “what is good” for all His Children. • Emergency baptism can be carried out by staff at the request of parents if the infant is at risk of dying before the arrival of a priest/pastor.

*Birgit (22), Caulfield et al. (6), and Lewis and Foley (21)*.

In a qualitative multicenter study, Boss et al. (5) described some parents whose infants had died due to severe prematurity or congenital defects as being reluctant to make any decisions regarding resuscitation. These parents were described as wanting matters “left … in God's hands,” yet typically they were documented to be most likely to “want everything done.” In this study, for most parents, faith, spirituality, and hope have influenced the decision-making process.

Prayer is an important component for Christians, regardless of the sub-denomination, and may be directed to one or all the holy trinity: God, the Holy Spirit, and/or Jesus Christ. Baptism, which is a Christian rite of entry into Christianity, almost always through the use of water, is another important ritual, usually performed near the time of birth. According to Birgit (22), the onus is upon parents to seek baptism if their infant dies at birth or is imminently dying as the unbaptized infant cannot “seek truth” or choose to behave in an “ethical way.” Christians believe that the baptized child who dies will end up in heaven, but there are reasons for hope that, in the presence of God, children who die without baptism will still feel the joy of everlasting life (i.e., enter Heaven) (22).

Caulfield et al. (6) have recommended that, in order to fulfill the needs of families in an emerging multi-faith community, maternity hospitals and neonatal units need to have access to emergency baptism services or other similar “spiritual blessings.” In a study to evaluate trends of infants of Catholic parents baptized over a 15-year period at an Irish maternity hospital, Caulfield et al. (6) reported a gradual decline of emergency baptisms.

Access to emergency baptism services is important, because, in the early stages of grief of parents whose infant had died, 37% of the parents (who were all Christian) said that their religious beliefs were challenged and that they questioned their faith (10). Parents in a study by Hawthorne et al. (10) expressed their anger and betrayal toward God (to whom they had prayed for help) for being cruel and unjust and the perception that they were being punished by His failure to protect their child; 9% said that they had lost their trust in God. While one of the most important early sacraments in Christianity is Baptism (22), it is important for clinicians to be mindful that many Christians may not subscribe to the point of view outlined in the study by Hawthorne et al.

#### Hinduism

Hinduism, with its origins and traditions dating back more than 4,000 years, is considered the oldest religion in the world. Hinduism is practiced by 15.1% globally and is a broad term that describes many subgroups of the religion, which influence its practice and customs. Most forms of Hinduism are henotheistic, adulating a single deity (*Brahman*), but other gods and goddesses are still acknowledged by their followers. In the scriptures and wisdom heritages of the Vedic tradition, however, all of them have their individual origins (8). Hinduism, irrespective of tradition, offers a systematic explanation of life and death that most Hindus accept. The term “life” translates to “*the atma*,” which can be interpreted as the “soul” (8). In Hinduism, achieving a state of *Nirvana* (or “oneness with God”) is the primary purpose. Similar to Buddhism, disease and illness are considered the result of karmic actions, and death is simply a passage because the atma has no beginning or end (21). Hindus believe in reincarnation, so a newborn is not a “new” creation but a perpetual being continuing its progression of lives in a new body.

In terms of infants born with an LLFD, the *Bhagavat Purana* (a revered Hindu text) affords personhood to the fetus at 180 days of gestation as the embryo begins to develop awareness and beings to feel pain (8). The *Dharmashastras* (the treatises of Hinduism) view the termination of pregnancy (the “killing” of a human fetus is referred to as “*bhrunahatya*”) as a sin; however, in less traditional Hindu families, termination may not be seen this way.

Clinicians should be aware that more importance is placed on the male child in Hinduism, given that they continue the lineage of the family, including the name. Therefore, it is more likely that the death of a male infant will be more acutely felt in traditional Hindu families than the death of a female infant (23). In the Hindu tradition, palliative care is considered appropriate for infants who are not expected to survive. Any interventions that might hasten death are not considered appropriate, and where possible, all active treatments should be ceased and the death of the infant should be allowed to happen in its own time (8). Table 5 provides guidance for clinicians caring for infants of Hindu families, especially for both the infant and the parents.

**Table 5:** Hinduism—what clinicians need to know.

**For the infant**	**For the parents and family**
• As in Buddhism, the illness of the infant is the result of past and current actions (karma). • Food is viewed as a gift from God and is often part of religious rituals. Not taking nutrition near the time of death is part of the preparation for the end-of-life. • The mother may pump and discard the colostrum, as it's believed to be unsuitable for infants (in Hinduism, most infants are fed sugar water instead.) • Avoid cannulation of the right hand/arm as the right hand is considered holy to promote self-cleaning. • The parents may wish to place holy water (from the Ganges) and basil leaves on the infant's body • Once deceased, the infant's arms should be straightened. • Parents may wish to tie sacred threads around the infant's wrists or neck. • Cremation is the norm in Hinduism. However, this norm generally doesn't apply to children who die within their first year. These infants are usually buried rather than cremated, or their bodies may be consigned to rivers.	• Hindus may place more importance on male children (due to the family name and lineage); the death of a male child may be more acutely felt than the death of a female child. • The father is the head of the household, and decision making is often made at the family leve.l • Hindus believe in reincarnation, believing their child may be reborn in the same family to their children or grandchildren and return to earth. • Death should be a peaceful transition from life to death, ensuring the soul will rest in peace. • Like most, Hindus view death of a child in any family as one of the worst misfortunes imaginable.

*Chichester and Wool (7), Jayaram (23), and Lewis and Foley (21)*.

#### Judaism

Judaism is practiced by <0.2% of the global population. Judaism is the oldest monotheistic religion, dating back nearly 4,000 years. The followers of Judaism believe in one God who, through ancient prophets, revealed himself. Its primary religious beliefs revolve around a code of ethics with four groupings of Jewish beliefs: reform (largest affiliation [35%] of American Jews), reconstructionist (an evolving civilization of the Jewish people), conservative (“traditional” Judaism outside of North America), and orthodox (adherence to a traditional understanding of Jewish law; this includes Haredi and Hasidic). Jewish clergy are known as *Rabbi*. Psalms, as the last prayer of confession (*vidui*), are conducted at the bedside of the person (21).

Table 6 provides guidance for caring for infants of Jewish families, especially for both the infant and the parents.

**Table 6:** Judaism—what clinicians need to know.

**For the infant**	**For the parents and family**
• Prayer shawls are common and are often passed between generations. • Food and water are considered basic needs. Withholding feeds may not be considered acceptable. However, it is appropriate to remove artificial nutrition and hydration to allow death to take its natural course if death is imminent. • Pain management is acceptable and preferable (and may be mandated) even if death is hastened. • At death, the infant's arms should not be crossed. • Where possible, any clothing or dressings with blood on them should also be prepared for burial with the infant. Jewish people believe it is important that the whole person be buried together. • Encourage the parents to see and touch their infant, photos are appropriate, and it is appropriate to collect mementos. • If the parents have not named their infant, it is appropriate to encourage them to do so. • Autopsy is appropriate (and even encouraged) if it can help determine the future pregnancies for the couple. Remember that the whole person needs to be buried, however. • The infant should be buried as soon as possible, in accordance with Jewish practice.	• All human life has value, principles such as quality of life may not contribute to the decision for curative or palliative care. • It was once thought that if the infant died before 30 days, including the full 30th day the infant is not mourned (Shulchan Aruch Yoreh De'ah 374:8), however nowadays this is mostly untrue. [The Yoreh De'ah is a section of Rabbi Jacob ben Asher's compilation of halakha, Arba'ah Turim]. • If the pregnancy was full-term, parents and other family members are generally obligated to have full bereavement practices, just as for any other child. • The parents will regard the healthcare team as being in a place of authority and may look to their doctors for their opinions regarding treatment options. • Following the death of the term infant, parents will recite Kaddish [hymn of praises about God found in Jewish prayers] for 30 days and may observe yahrzeit [the first anniversary of the death]. • In the case of premature birth, there is still debate within the Jewish community on how to define “viability.” Consult with parents and their Rabbi to guide decision making. • No work can be done on the Sabbath (Friday sunset to Saturday sunset). This may result in parents delaying important decisions, including driving to the hospital to speak with the health care team.

*Chichester and Wool (7) and Wolfson (24)*.

A prematurely born infant is referred to as a “*Nefel*”: the same word is often applied to an abortus or an infant born with major life-incompatible defects. The word Nefel, in Jewish culture, refers to an infant who lacks sufficient hair and nail growth; is born in the eighth month of pregnancy; and survives for <30 days. Relevant social rules are different for a Nefel in Judaism compared to a well term infant (12).

It was once thought that if the infant dies, up to and including, on the full 30th day, then the infant is not mourned (Shulchan Aruch Yoreh De'ah 374:8); however, nowadays this is mostly untrue. [The Yoreh De'ah is a section of Rabbi Jacob ben Asher's compilation of halakha, Arba'ah Turim]. It has been reported that Jewish parents still feel a keen sense of loss and grief when an infant dies before the thirty-day benchmark (24).

### Spiritual considerations for parents

Though used interchangeably, faith and spirituality are different from each other. Spirituality is more difficult to define because there are no common characteristics that were agreed upon. It is a broader concept that each individual defines for themselves (18). Spiritual care is distinctly different from identifying and resolving specific health problems: It is about being present in the journey of “making meaning” at the pivotal times in one's life.

In an explorative study about relevant sense-making activities, whether through prayer and/or meditation, first-time mothers established two key themes to help them deal with the life-threatening illness of their infants: (a) looking for meaning circles and (b) using intuitive/spiritual experiences to find meaning (13). Similarly, Sadeghi et al. (3) found three spiritual themes identified by parents that had lost an infant following an LLFD: a philosophical belief in the forces of the supernatural; the need for the comfort of the soul; and human dignity for the newborn (3).

To help parents make meaning and meet their spiritual needs when faced with perinatal death, the parents should be encouraged to consider how their infant has touched other lives besides their own; this includes other family members, particularly grandparents, who would have had their own hopes and dreams for the child. Raingruber and Milstein (13) propose that parents imagine the waves that circle around just one pebble tossed into a pool and that this enables parents to recognize how the life and/or death of their child form others. Parents will gain an insight into the life and death of their baby by focusing on these waves (13). These simple practices do not require parents to follow a particular religion, and comfort can be found in simple acts of making meaning. It is important to support parents through their loss by providing care that also addresses the spiritual, religious, and, existential needs of the parents and families (2).

In 2017, Hawthorne et al. explored the disparities between gender, race/ethnicity, and religion in the use of spiritual and religious coping strategies by parents after the death of a child. The study found that non-Hispanic black mothers were more likely to use religious coping strategies than non-Hispanic white mothers. Christian parents used more religious coping practices than the other groups, and mothers reported considerably more use of religious and spiritual coping practices than fathers (10). This study concluded that black mothers were more religious than they were spiritual, and the mothers were more spiritual and religious than the fathers in coping with the loss of an infant, providing some guidance to healthcare professionals on how to support bereaved parents of diverse races/ethnicities and religions. It has been identified in Latino women that their religious and spiritual beliefs were found to be intensely comforting. Their combined beliefs helped them to validate the decisions they had made in response to an LLFD. Their spirituality helped them to maintain a sense of optimism even when faced with difficult decisions following complex amniocentesis findings (14).

### Cultural Considerations for Parents

Culture refers to the collective deposit of information, experience, opinions, principles, behaviors, meanings, hierarchies, faith, notions of time, responsibilities, spatial relationships, concepts of the world, and material objects and belongings acquired through person and community by a group of people over different periods of time (25).

In 2008, Davies et al. conducted a survey demonstrating that almost 40% of healthcare professionals cited “cultural differences” as a barrier to providing palliative care. This was particularly so among ethnic minorities whose cultures were poorly understood, including Latino, Indian, and Native and African Americans. Cultural gaps extended to language barriers, religious differences, and mistrust of healthcare providers and care of health practitioners who did not have the same racial or cultural context and who did not recognize these differences (26). In a study that explored the challenges for trainee neonatologists, dealing with different cultures and the inability to interact effectively with people of different cultures were identified as significant when the trainee had different faith and religious beliefs to those of the parents and families. The trainees were more likely to respond with anxiety, wariness, and even anger or fear to unknown cultures or unfamiliar cultures when working with parents that are making the end-of-life decisions about their infant (9).

No matter how committed, highly trained, and dedicated the healthcare team is, when caring for parents and their infant with an LLFD, an expert understanding of how families function, spiritually, cross-culturally, and existentially, presents a considerable challenge in PPC.

## Conclusions

It is not surprising that families rely on their faith and spirituality in times of crisis to help them make sense of the path to the end of life. This analysis revealed why healthcare practitioners need to become familiar with the definitions of the moral and religious ideas that arise in their clinical field or specialization for parents and families. Regardless of the religious, spiritual, or cultural beliefs, all parents facing the death of their infant due to an LLFD require a compassionate, supportive, and knowledgeable healthcare service. Life and death are momentous occasions in the life cycle, and when the two occur in close proximity, beliefs and religious ceremonies become even more profound. All members of the healthcare team providing care to parents from the moment of the diagnosis through the palliation of to death of the infant require a unique skill set, including the ability to pay attention to, and the knowledge of, religious, spiritual, and cultural needs of the parents.

## Author Contributions

VK conceptualized and designed the review, drafted the manuscript, and is solely responsible for data collection and interpretation of the literature data.

## Conflict of Interest

The author declares that the research was conducted in the absence of any commercial or financial relationships that could be construed as a potential conflict of interest.
